# Connectivity, Cycles, and Persistence Thresholds in Metapopulation Networks

**DOI:** 10.1371/journal.pcbi.1000876

**Published:** 2010-08-05

**Authors:** Yael Artzy-Randrup, Lewi Stone

**Affiliations:** 1 Department of Ecology and Evolution, University of Michigan, Ann Arbor, Michigan, United States of America; 2 Howard Hughes Medical Institute, University of Michigan, Ann Arbor, Michigan, United States of America; 3 Biomathematics Unit, Faculty of Life Sciences, Tel Aviv University, Tel-Aviv, Israel; Estación Biológica de Doñana, CSIC, Spain

## Abstract

Synthesising the relationships between complexity, connectivity, and the stability of large biological systems has been a longstanding fundamental quest in theoretical biology and ecology. With the many exciting developments in modern network theory, interest in these issues has recently come to the forefront in a range of multidisciplinary areas. Here we outline a new theoretical analysis specifically relevant for the study of ecological metapopulations focusing primarily on marine systems, where subpopulations are generally connected via larval dispersal. Our work determines the qualitative and quantitative conditions by which dispersal and network structure control the persistence of a set of age-structured patch populations. Mathematical modelling combined with a graph theoretic analysis demonstrates that persistence depends crucially on the topology of cycles in the dispersal network which tend to enhance the effect of larvae “returning home.” Our method clarifies the impact directly due to network structure, but this almost by definition can only be achieved by examining the simplified case in which patches are identical; an assumption that we later relax. The methodology identifies critical migration routes, whose presence are vital to overall stability, and therefore should have high conservation priority. In contrast, “lonely links,” or links in the network that do not participate in a cyclical component, have no impact on persistence and thus have low conservation priority. A number of other intriguing criteria for persistence are derived. Our modelling framework reveals new insights regarding the determinants of persistence, stability, and thresholds in complex metapopulations. In particular, while theoretical arguments have, in the past, suggested that increasing connectivity is a destabilizing feature in complex systems, this is not evident in metapopulation networks where connectivity, cycles, coherency, and heterogeneity all tend to enhance persistence. The results should be of interest for many other scientific contexts that make use of network theory.

## Author Summary

Taking advantage of modern network theory, we present a model formulation for determining those factors that control the stability and persistence of complex biological systems. As a case study, we focus on ecological metapopulations, which may be viewed as a set of distinct subpopulations (/sites) that are connected via a dispersal network of arbitrary complexity. Metapopulation persistence is found to depend critically on the topology of cycles, and cyclical components in the connectivity network, because they allow the offspring of the population to eventually “return home” to the sites from which they originated. The methodology identifies critical migration routes, whose presence are vital to overall stability, and are thus of high conservation priority – information that may be of value when designing networks of marine protected areas. In contrast, links that do not participate in a cyclical component have no impact on persistence and thus have low conservation priority. While network theory is highly fashionable in biology, only few studies go deeper than descriptive statistical applications as attempted here. Moreover, the key results are easily extended to other biological contexts (e.g., disease networks), particularly in situations whereby the network controls the dynamics of a complex system.

## Introduction

Theoretical biologists and ecologists have long sought to understand the relationships between complexity, connectivity and the stability of large biological systems [1]–[10]. This interest has only grown in recent years particularly with the new developments in modern network theory and its multidisciplinary applications [11]–[15]. Here we outline a new synthesis relevant for ecological metapopulations, and provide a framework for untangling the role of dispersal and network structure in maintaining the persistence of a set of patch populations distributed in space. The metapopulation concept has become the theoretical framework that stands behind many modern conservation efforts [2]–[4], [16]–[29]. For example, the framework was adopted as part of the EU Habitats Directive Natura 2000 [20], which is the single most important legal tool for biodiversity conservation that has become binding national law of all European Member States. The Directive's goal is the creation of a coherent Europe-wide network of sites to protect important habitats and species. Ecological coherence “is seen in terms of the capacity for individual protected areas to support each other and in the interactions with habitat surrounding protected areas.” Species dispersal between sites provides supportive buffering for impacted habitats (e.g., oil spills) or allows for shifts in species ranges in the face of climate change, thus enhancing overall coherence. Similar metapopulation approaches have been made use of in the development of Marine Protected Areas (MPA's) – areas of the ocean protected from human disturbances – and they are currently strongly advocated as a tactical management tool [20]–[22].

Given the importance of the metapopulation concept for nature conservation, theoretical studies have attempted to gain insights into those factors that make for sustainable populations. Intriguingly, initial research indicated that spatial structure has in fact very little effect, with criteria for metapopulation stability appearing to be identical to the stability conditions for a single patch [23]. Several important studies have shown that variability or heterogeneity in patch connectivity may play a role in enhancing persistence [24], [25], but this work has not been developed further. Interest in these issues has resurfaced in recent years especially in the study of marine ecosystems where connectivity and the role of dispersal in maintaining persistent metapopulations is as controversial as it is enigmatic. Whether marine populations are retentive and recruit back to their native populations, or whether they are open and in the main, disperse with little self-recruitment, is an issue that remains unresolved despite decades of work. As yet, there is little understanding of how the architecture of marine networks control metapopulation persistence, and even fundamental concepts still remain controversial. Hastings and Botsford [3] concluded that multiple criteria are necessary to assess persistence, which explains why they were unable to “obtain a single number [criterion] like the reproductive number for a single population.” Here we derive simple general persistence criteria that are couched in terms of the metapopulation's reproductive potential, network connectivity as well as the topology of cycles in the dispersal network.

Similar to [3], we consider populations that comprise sedentary adults with dispersing juvenile stages, making the model appropriate for marine and terrestrial invertebrates, as well as many plants and fish. However, unlike most other studies, the model includes age-structure and may be generalized further to allow for dispersal across patches between the population's different age-classes. The model is particularly suited to marine systems where questions concerning connectedness, larvae retention and open versus closed populations have become highly topical in the last years.

We begin by considering a population that has *m* age-classes as described by the vector 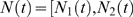 where 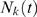 is the number of individuals in the *k*'th age class in year *t*. For such a population in a single isolated patch, growth may be modelled via the familiar Leslie matrix equations [31]:
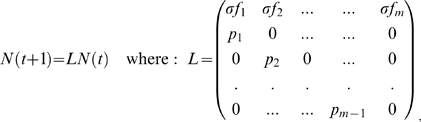
(1)and the time-step is one year. Here 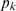 is the probability that an individual of age (*k*-1) years survives to age-*k*. A fraction 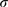 of juveniles from the population successfully “self-recruit” and return to the population after the dispersal phase, which in marine settings might represent local larvae retention. The parameter 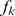 *r*epresents the fertility of age-class-*k* individuals, in terms of the average number of juveniles produced in the next generation. A graph theoretic interpretation of more complex stage-structured Leslie matrix population models may be found in [32].

The population has a single equilibrium, the extinction state 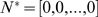 whose stability is the basis for understanding population persistence in this model. It is a classical result [2], [31] that the average number of juveniles produced in the lifetime of a typical individual is given by the reproductive number:
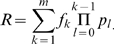
(2)The condition for population persistence (i.e., a growing population) requires that a typical individual is capable of replacing itself and give rise to at least a single offspring that successfully recruits back to the population. As only a proportion 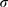 of juveniles successfully self-recruit, the net reproductive effort is 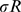. Thus a growing persisting population requires that the persistence parameter:
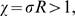
(3)and the patch is said to be a “source.” Should 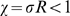, all age-classes approach a stable extinction state and the patch is said to be a “sink.” These definitions of source and sink follow those used in Armsworth [2] and are based on the original definitions of Pulliam [30], although it should be noted that other definitions are sometimes employed. In words, a sink would correspond to a situation where deaths and emigrations exceed the number of new juveniles [2]. A mathematical analysis of the model's single equilibrium, the extinction state 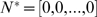, corroborates the above threshold criterion.

**Figure 1:**
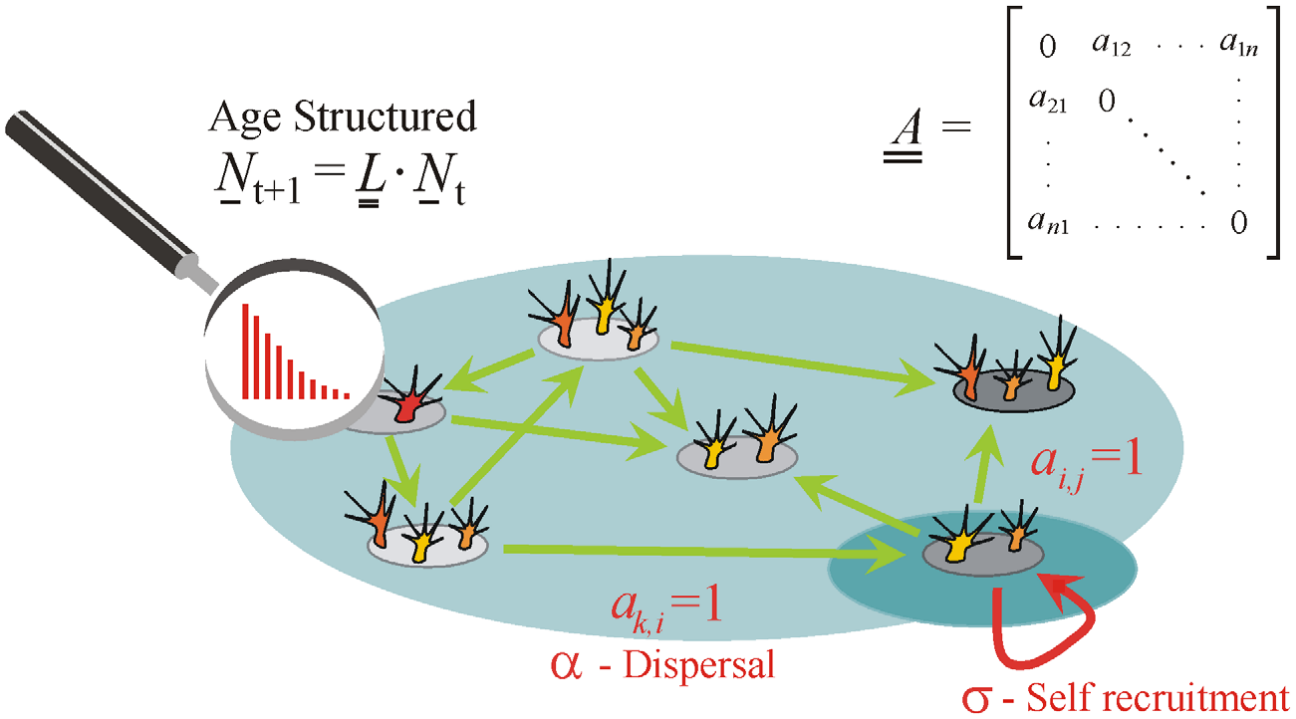
The topology of the dispersal pathways between a set of patches as defined by the adjacency matrix 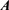. The parameter 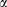 represents the intensity of dispersal between patches while σ represents the amount of self-recruitment in each patch. The population dynamics within a patch follow the Leslie matrix growth equations 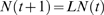 where 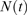 is a vector defining the numbers of the population in each age-class at time *t* while the matrix 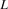 defines survival and fertility rates.

Now scaling up, consider a network of *n* age-structured patch-populations, where juveniles disperse between patches as portrayed in Figure 1. The metapopulation dynamics are given by:
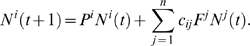
(4)In this notation 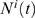 is the *m*-dimensional age-class population vector at patch-*i*, and each patch has its own associated survival (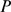) and fertility (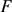) matrices. More specifically:
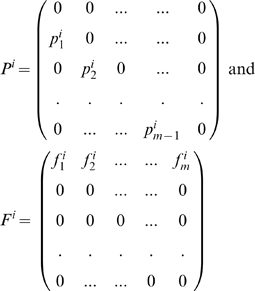
(5)where the Leslie matrix 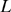 in Eqn.1 is retrieved taking 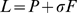. Dispersal processes between the *n*-patches are defined in the connectivity matrix 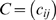 whose elements 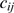 correspond to the proportion of juveniles produced on local population-*j* that are transported and successfully recruit to local population-*i*. We note that Eqns. 4 may be viewed as a good approximation to a more complex nonlinear metapopulation model (e.g., possibly with density-dependence), if the latter model is linearized about its extinction state. The asymptotic stability criterion for the extinction state is then the same for both the linearized and full nonlinear model.

## Results

### Connectivity thresholds

A key goal is to examine the effects solely of network architecture on metapopulation persistence. This requires studying the metapopulation with all processes being equal apart from the network structure. We thus initially suppose that local patch populations are identical, and all with the same survival and fertility rates 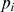 and 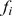, an assumption that is later relaxed. Based on the properties of the single patch dynamics noted above, it is possible to deduce (see Materials and Methods, as well as Text S1) that metapopulation persistence is controlled by a simple threshold condition: The metapopulation is growing if the fundamental persistence parameter
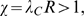
(6)while the extinction state is stable if 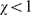. Here 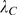 is the spectral radius, or eigenvalue of largest magnitude, of the connectivity matrix 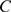. As before, 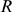 is the reproductive number of a single patch as defined in Eqn. 2. The special role played by the matrix 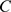 allows us to view the condition as a connectivity threshold. Although previous studies of age-structured metapopulations [2], [4], [21] attempted to deduce persistence criteria, they overlooked the role of the spectral radius 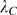.

The metapopulation's complex patch dispersal structure may be visualized in terms of a network or graph, with nodes as patches and edges as dispersal links between patches. The network topology is summarised by the adjacency matrix 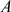 whose elements 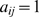 if there is a direct dispersal route from patch-*j* to patch-*i*, while 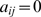 in the absence of such a route. In the formulation used here self-loops are excluded from the adjacency matrix, and we can set 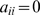.

Again, in order to specifically elucidate the effects of network structure, it is necessary to ensure that the various processes between patches are kept equal. This motivates a relatively simple but nevertheless useful scheme in which the number of juveniles that immigrate to a patch population is on average a fixed proportion, 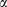, of the source population from which they originate as shown in Figure 1. The average retentivity, or self-recruitment, of each patch is set at 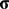 indicating the proportion of juveniles that complete their life-cycle in the patch. In this scheme highly connected patches recruit better, while less connected patches are disadvantaged with recruits being lost from the metapopulation as a result. Such losses are to be expected in a consistent model of heterogeneous dispersal.

The connectivity matrix may now be written in the particularly simple form: 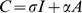 where 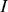 is the identity matrix. This immediately yields the key relationship whereby the persistence parameter (Eqn. 6) may be written as:
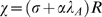
(7)We see directly that persistence is controlled by 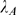, the spectral radius of the adjacency matrix 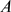. We note that it is not always a simple matter to deduce the value of the spectral radius without resorting to a numerical study of the 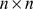 adjacency matrix 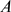. Factors that control the spectral radius include the number of nodes (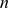), the number of edges and the underlying structure of the matrix in terms of the topology of the dispersal routes. However, there are special cases that we will examine, where the particular structure of 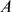 makes it possible to solve for the spectral radius analytically.

The expression (Eqn. 7) for the persistence parameter shows clearly the importance of supply-side ecology with new recruits enhancing the possibility of persistence. A metapopulation, in which all *n* patch populations are sinks (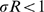) requires sufficient subsidy recruitment (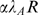) for persistence; enough that ensures 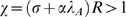. In such a case, the metapopulation is effectively “pulled up by its bootstraps” due to the inflow of larvae circulating through the complex dispersal routes of the network. The additional recruitment here acts as a rescue effect.

**Figure 2:**
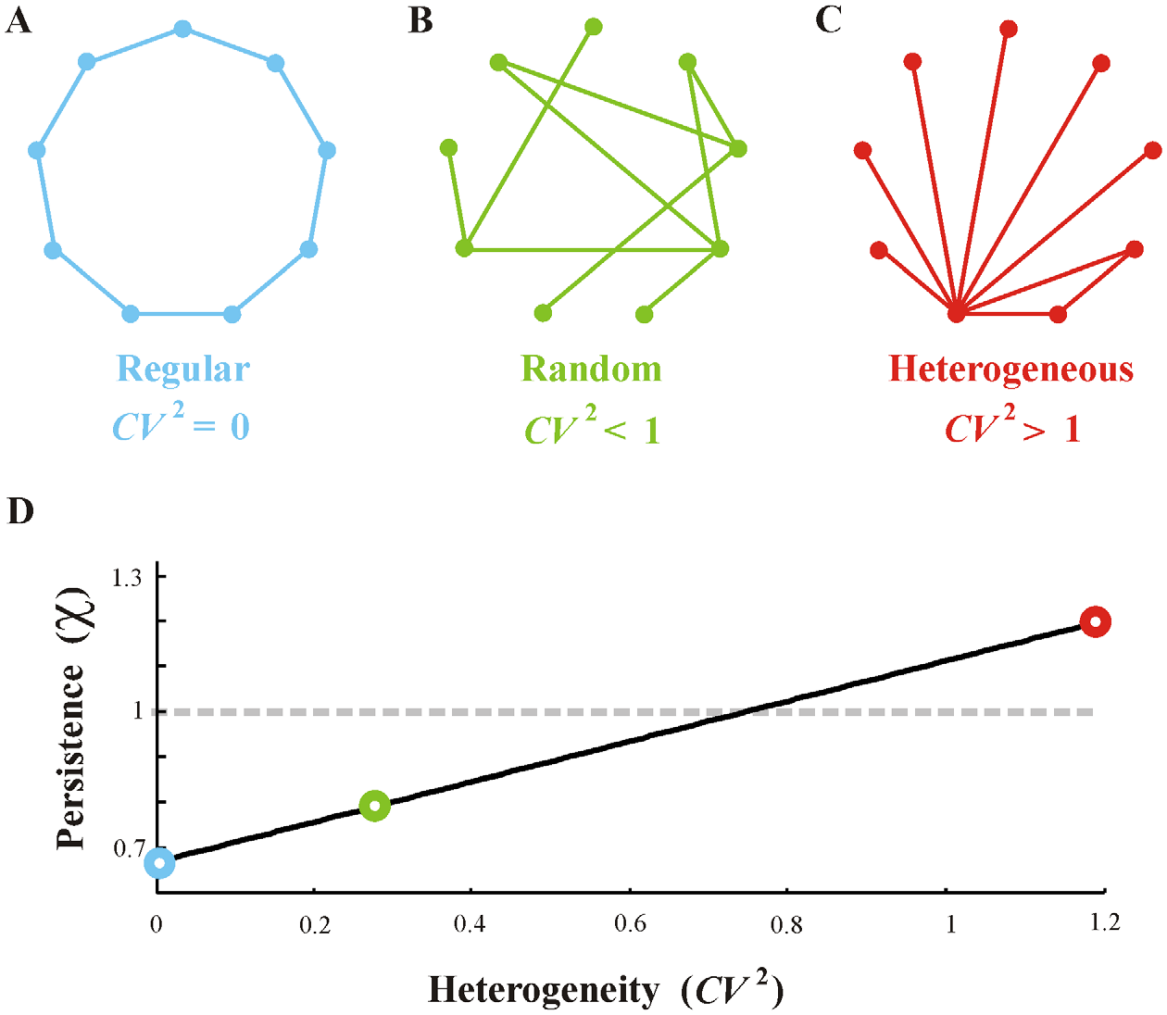
Persistence as a function of heterogeneity in node degree for three example networks. A) Regular, B) Random, and C) Heterogeneous networks, all with mean degree of 2. D) The persistence parameter 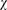 is calculated as a function of 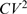 according to Eqn.9 with parameters: 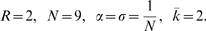

An exciting outcome of Eqn. 7 is that it opens the door for investigating the effects of network structure, at both fine and coarse-scale levels. Beginning with coarser-scale features, we focus on network topology. Figure 2 shows three simple network topologies: **a)** a regular network in which each patch has exactly two connections; **b)** a random network in which patches have very close to two connections; **c)** a random heterogeneous network in which patches have large variability in connectivity. For all three networks the average number of connections per patch, or mean degree, is deliberately held the same.

It is then natural to ask which topology best enhances persistence and how does the topology's heterogeneity affect the threshold 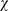
? This is far more than an academic question given that marine systems can be highly heterogeneous in terms of recruitment, sometimes with orders of magnitude variation to patches within the same metapopulation [33].

The three above cases are now treated separately assuming the networks are undirected; that is assuming a dispersal pathway from patch-*i* to patch-*j* implies a converse flow from patch-*j* to *i*. However, the results obtained are substantially similar with interpretations that broadly carry over to the case of directed networks [34].

#### a) Regular networks

In a regular network every patch is connected to 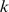 other patches (see Figure 2a). Again we suppose each patch is recruiting equally from every other, but now with individual migration rates equal to self-recruitment rates 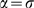. Since it is well known for regular networks that the adjacency matrix 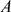 has spectral radius 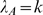 [34], then metapopulation persistence is ensured if 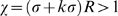. This is illuminating in that it shows that the gain of an individual patch derived from subsidy-recruitment (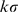) can be considerably larger than self-recruitment (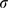), and that this “bootstrapping” contribution may be of significance for crossing the persistence threshold.

#### b) Random networks

The classical Erdős–Rényi (ER) random network assumes there is a probability 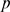 that any two patches -*i* and -*j* have a dispersal pathway between them (Figure 2b). As the network has 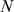 patches, the mean number of dispersal pathways per patch is 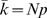. For these random networks, it is well known that the spectral radius of the adjacency matrix 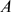 is 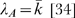 [34] and:
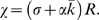
(8)Thus increasing connectivity simply by the random addition of dispersal routes into a metapopulation increases the mean degree 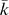, and thus 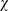, and should therefore be seen as advantageous. The more random pathways, the larger is 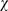, and the more likely is the possibility of increasing beyond the persistence threshold. This result has important consequences for the ongoing debate as to whether highly connected ecological and biological systems are more stable. Unlike May's [1] prediction where connectivity is seen to be destabilizing in large complex systems, here increasing connectivity clearly enhances metapopulation persistence. It should however be noted that strictly speaking May was referring to species interactions (although see [29]), while our study concerns something quite different, namely network connectivity.

#### c) Heterogeneous networks

The result can be generalized for heterogeneous random networks having arbitrary degree distribution (Figure 2c). Suppose patch-*i* has 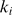 dispersal connections to other patches. The variability of the different 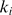 (i.e., the “degree distribution”) is a good index of the heterogeneity in connectivity of the network and is often measured by the squared coefficient of variation 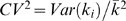. Under these conditions,
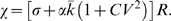
(9)Thus increasing the heterogeneity in connectivity (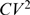) will enhance persistence (even if the mean degree 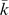 remains unchanged) since it increases 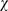 (see Figure 2d). Analogous results were found in [24]–[26] although not presented in the context of network heterogeneity as here. Curiously, large heterogeneity may be the norm in many metapopulations. Williams and Sale [33] found orders of magnitude variation in recruitment to patches of the same coral species in a single coral lagoon. In some cases substantial variation existed between patches 1km apart. Since then it has been shown that wildly fluctuating recruitment success is not particular to reef fish.

### Cycles

Finer-scale network features also play an important role in determining persistence. The present framework allows exploration of how various network motifs [8], [13] or specific subgraphs of the network, might be influential. The following analysis of cyclical motifs indicates the power of this approach. We focus on cycles in directed networks where a dispersal route from patch-A to B (A→B) does not necessarily imply the converse. Typically a closed loop for three patches would have the following structure A→B→C→A, and is termed a directed cycle.

#### C1) A metapopulation network without any cycles is unable to persist

Firstly, it becomes trivial to show that a metapopulation without any cycles in the dispersal network is unable to persist, as might be expected (see also [2]). In such a metapopulation network, all juveniles or any of their eventual descendants are unable to recruit back to their patch of origin – they never “return home” [3]. When there are no cycles, the adjacency matrix 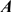 must have spectral radius 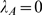 [35] and in the absence of self-recruitment (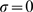), the persistence parameter 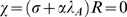. A stable extinction state is expected.

#### C2) For metapopulations with self-recruitment but without any other cycles, there is no advantage to dispersal

Self-recruitment 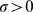 implies that some proportion of juveniles complete their life-cycle in the patch. As there are no other cycles, all other juveniles fail to return home and the spectral radius remains 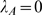 and 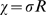. Thus the criterion for the entire metapopulation to persist, 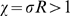, is precisely the same as the criterion for a single self-recruiting patch to persist (Eqn. 3). Consequently there is no advantage for dispersal if there are no cycles and larvae fail to “return home.”

#### C3) “Lonely links,” those links in a network that are not part of a cycle, have no effect whatsoever on the metapopulation's persistence characteristics

**Figure 3:**
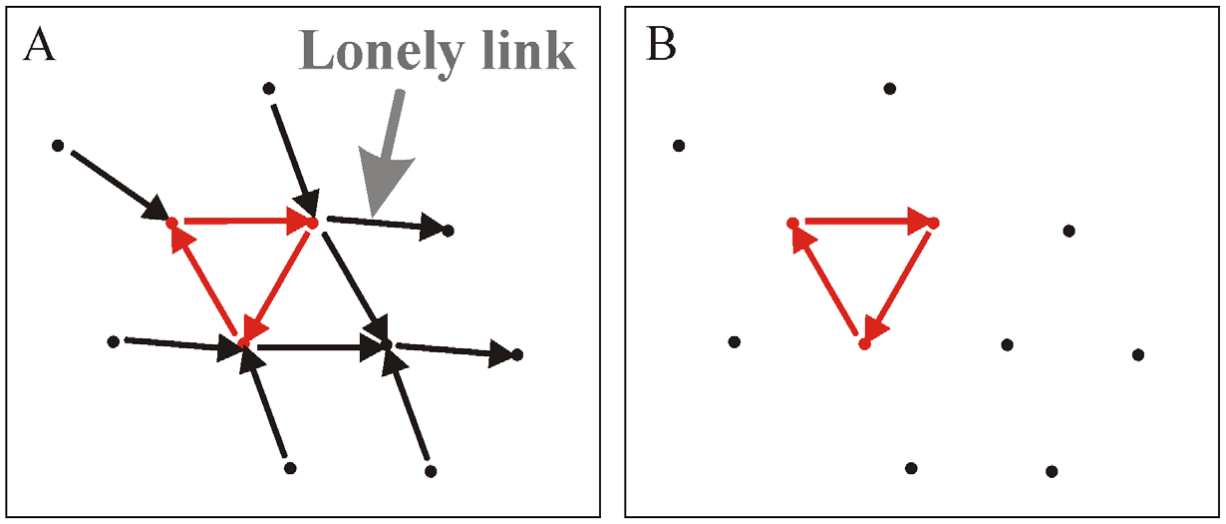
Example of removing lonely links. (A) A complex network with a single simple cycle (red). Eliminating all “lonely links” that do not belong to any cyclic component results in the network shown in (B).

Figure 3a illustrates a complex network with a single simple cycle (red), or cyclic component (see Text S2 as well). Eliminating all lonely links that are not connected to the cyclic component results in the network shown in Figure 3b. As proven for the general case (see Text S2), the persistence parameter 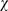 associated with the two networks is identical and hence both have the same persistence/stability properties. In short, the removal of “lonely links” has no effect on metapopulation persistence making them of low conservation priority.

#### C4) The persistence parameter 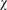 increases with the complexity and richness of the network's cycle structure

Consider first simple cycles, with no sites receiving recruits directly from two distinct cycles. Directed networks composed of only simple cycles, have the property that 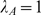 and thus 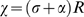, regardless of how many simple cycles are present. If cycles intersect they are no longer simple and in general 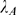, and thus 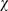, increases as the number of overlapping cycles increases. Intuitively one notes that maximal “cycle packing” might be considered analogous to a regular network with all *n*-patches connected to each other giving 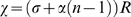. Thus metapopulation persistence is greatly enhanced when there is intense overlapping of dispersal routes between sites in the network.

#### C5) Networks may be broken down into nonoverlapping cyclic components

**Figure 4:**
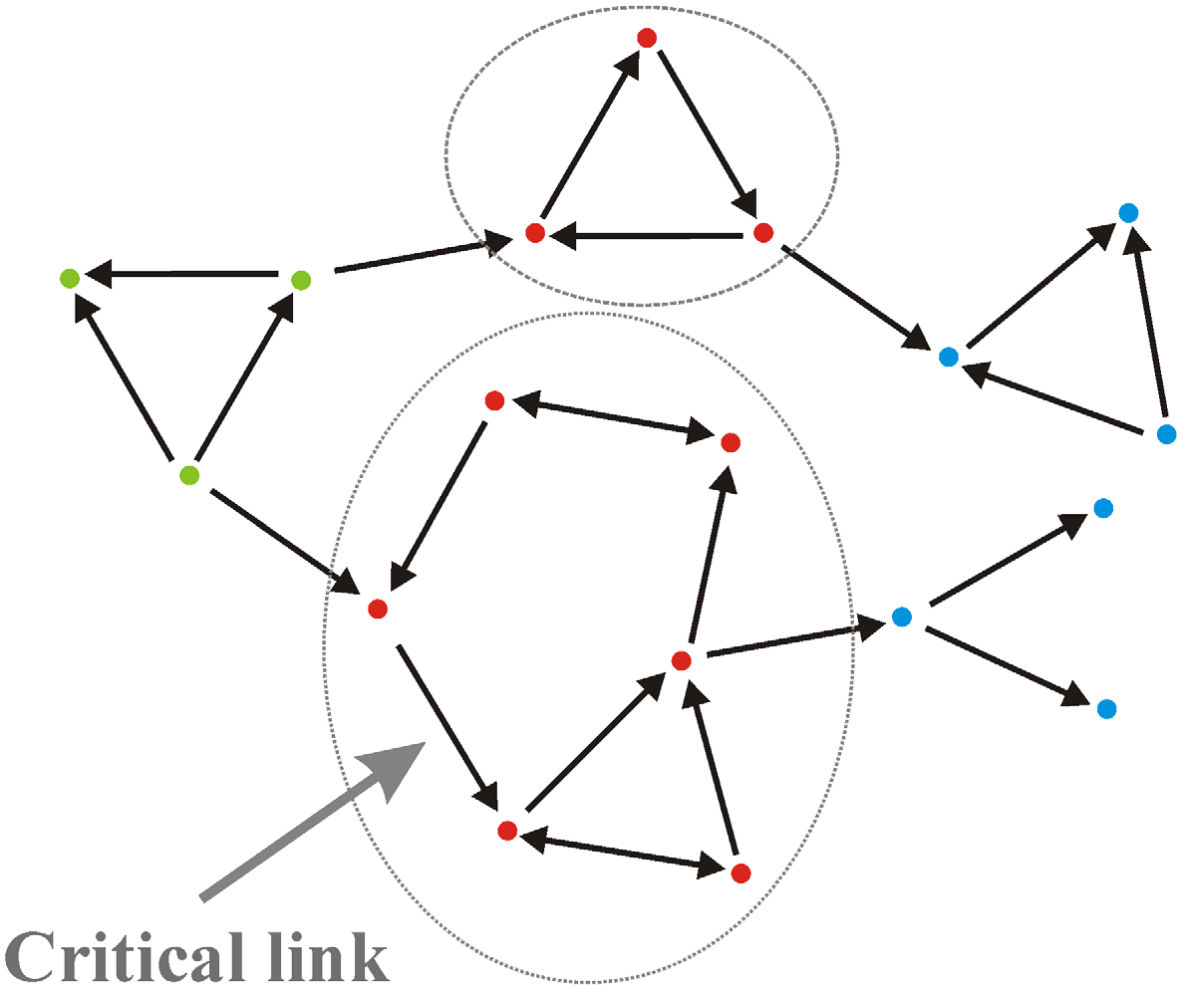
Persistence is controlled by the network's cyclical components of which there are two here (red patches); one simple component (3 patches, 3 links) and one complex component (6 patches, 9 links) formed by intersecting cycles. The other nine patches (blue, green) may be completely neglected since they do not belong to any cycle and therefore play no role in determining persistence. The characteristic equation for the eigenvalues of the adjacency matrix 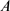 is (see Text S2 for details):
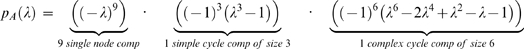
The spectral radius (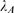) of the adjacency matrix 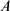 is the root of largest magnitude of the polynomial arising from the complex cyclical component: 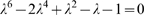 (giving 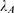). Thus the complex component exclusively controls persistence in that it alone determines the spectral radius of the adjacency matrix 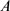. The new characteristic equation after removal of this link is:
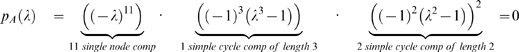

Persistence, is completely determined by the dominant cyclic component. Intriguingly it turns out that valuable information regarding persistence may be obtained by breaking down a network into its nonoverlapping cyclic components. In particular, we are able to show (Text S2) that persistence is completely determined by the dominant cyclic component, that is, the component whose associated eigenvalue is largest in magnitude. The complex network shown in Figure 3c illustrates this concept. Stripping away all lonely links, reveals that the network has two cyclic components (encircled). Extracting the larger eigenvalue associated with the more complex cyclic component, gives us 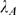 and persistence may be checked using [Eqn. 4] (see Text S2). Thus the dominant cyclic component gives complete information concerning the prospects of metapopulation persistence. Note that while “lonely links” have no effect on metapopulation persistence, the example in Figure 4 shows that the existence of a single critical link may in some cases determine the fate of the entire system. Thus removal of the dispersal route (see arrow pointing to critical link in Figure 4) in the dominant cyclic component can reduce 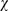 dramatically, and persistence will be lost. More specifically, removal of the critical link indicated reduces the spectral radius of 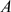 by approximately 33% (from ∼1.5 to 1) and thus reduces the persistence parameter 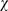 considerably possibly resulting in extinction. As such this route is of considerable importance from the perspective of conservation.

#### C6) Symmetry

It is revealing to return to the earlier examples of Figure 2a–c that deal exclusively with undirected symmetric networks. As dispersal is often viewed as a function of distance rather than direction, symmetric connectivity is a common assumption in metapopulation models [36]. Yet recent research has revealed that ecological and metapopulation networks are often asymmetric, [7], [37] and according to simulation studies in [7], [36]–[38], this should have a negative effect on persistence. The framework advanced here provides a simple theoretical explanation, based on the elementary observation that symmetric networks tend to have a propensity of cycles. Even a single connection between two patches in a symmetric network is bidirectional (A→B and B→A) and thus forms a cycle. Increasing asymmetry, tends to remove cycles, and by property C4 reduces 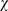, thereby having a negative effect on persistence. Text S2 delves into these properties further.

### Extensions: Nonidentical patches

So far, persistence has been discussed in the context of networks of identical patch populations. However this work may be extended to obtain persistence/extinction criteria for metapopulations comprised of nonidentical patch populations thus accommodating cases where age structure varies between patches. For convenience, suppose patch-1 of the metapopulation “dominates” in the sense that it has the highest fertility parameters (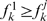 for all 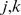) and highest survival rates (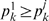 for all 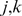) (see Text S3), and thus the highest net reproductive effort (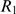). Now construct a metapopulation comprised of *n*-identical copies of dominating patch-1, retaining the original directed or undirected network structure. Test whether the extinction state of these *n*-identical patches is stable. If so, it can be shown that the original heterogeneous metapopulation of *n*-nonidentical patches is also unable to persist (see Text S3).

**Figure 5:**
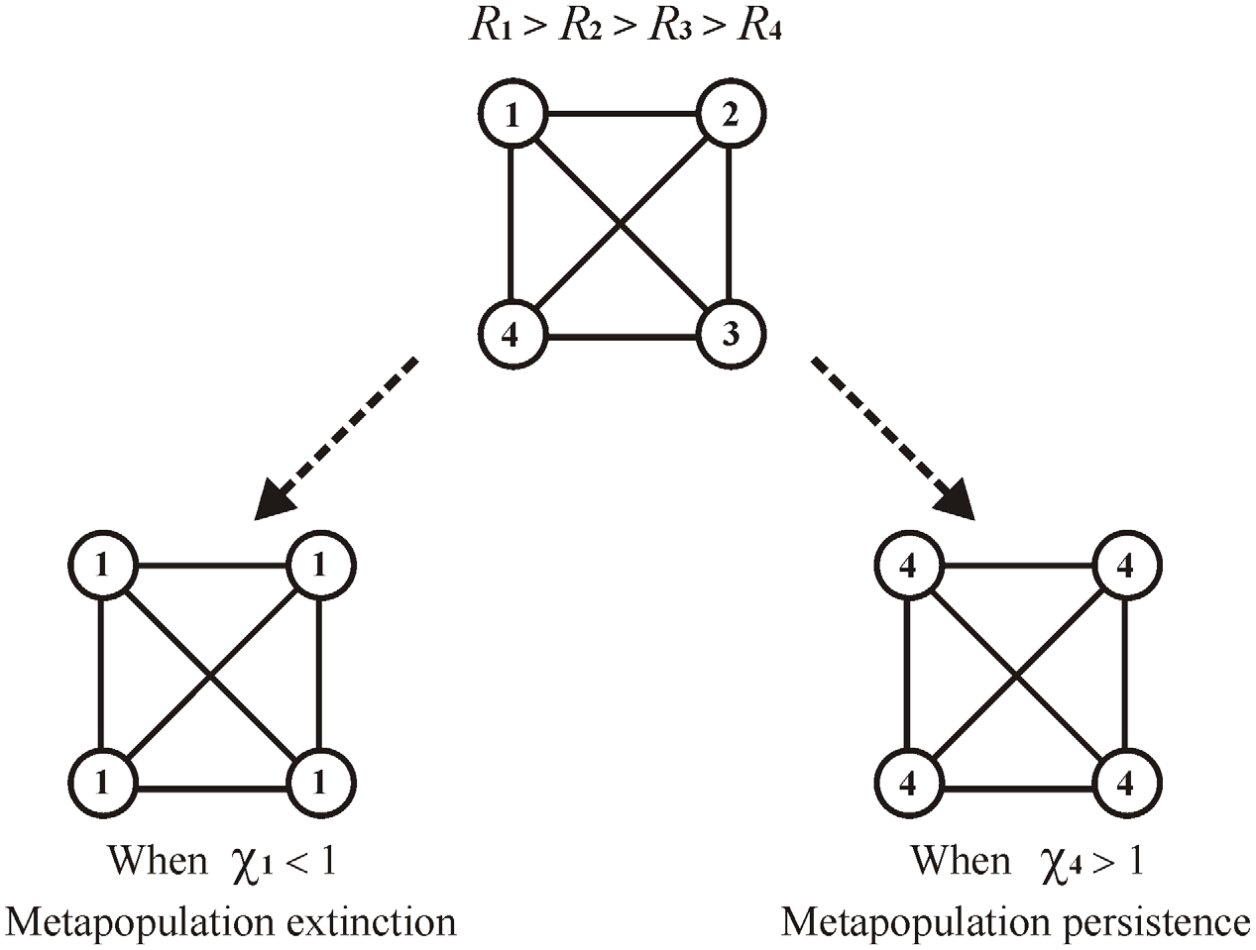
Metapopulation of four nonidentical patch populations in an “all to all” network. The reproductive number of the patches are such that *R*_1_>*R*_2_>*R*_3_>*R*_4_. Bottom left: to test for extinction, construct a hypothetical metapopulation of *n*-identical copies of dominating patch-1, but retaining the original “all to all” connectivity structure. If the hypothetical metapopulation has 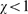, the original metapopulation on the top will also have a stable extinction state. Bottom right: to test for persistence, construct *n*-identical copies of patch-4, retaining the original connectivity structure. If the hypothetical metapopulation has 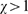, the original one on the top will also persist.

Along similar lines, suppose patch-1 is the weakest in the sense that it has the lowest fertility parameters and lowest survival rates. If the network of *n*-identical copies of patch-1 is able to persist, then the original heterogeneous metapopulation must also persist. Thus, the dominant and subordinate patch population of a network, may be used as a guide for determining bounds for the respective extinction or persistence thresholds of the larger heterogeneous metapopulation. These effects are illustrated in Figure 5 and discussed in Text S3.

It should be noted that the effects of age-structure become prominent with more complex migration schemes, for example, between different age-classes from different patches, and representations of ontogenetic shifts in habitat use that are life history dependent [[21]; and see Text S3].

## Discussion

Our work has shown the importance of the persistence parameter 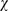 in assessing the fate of the metapopulation. As has been emphasized, a persisting metapopulation is characterized by a state of growth with 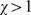, and implies that at least one patch population, say patch-1 of Figure 5, is increasing in numbers with time. This raises the question as to *which other patches in the metapopulation persist, and which go extinct?* Given that patch-1 increases in time, it must also continuously export larvae to all other patches it connects to, either directly or indirectly. Thus every patch that can be reached by patch-1 via a chain of dispersal pathways will continuously receive new recruits, and must therefore also persist, irrespective of whether it is a sink or source. In contrast, patch populations that fail to receive new recruits because they are disconnected from other patches in the network, cannot persist if they are sinks. This has the important implication that the underlying architecture of the metapopulation network controls the particular set of patch populations that survive (see Figure 4).

Ideally, we also require conditions that establish metapopulation *coherency*, whereby not only subsets of patches persist, but they are also connected with one another directly or indirectly in a large-scale manner that connects together the majority of the metapopulation network. Coherency offers the metapopulation many advantages. Large scale or global dispersal between sites buffers population fluctuations [39], allows recolonization after local isolated environmental impacts (e.g., oil spills), allows species ranges to shift in the face of climate change [20] and more generally allows “spreading of risks”; properties which are all verified from stochastic metapopulation simulations. Note that it is assumed that dispersal is not strong enough to induce large-scale spatial synchronization, a phenomenon that is prone to enhancing extinction risk [40].

The random Erdős–Rényi (ER) model which exemplifies a broad range of complex networks shows the existence of important coherency thresholds that must be taken into account. Recall that there is a probability *p* that any two patches-*i* and *j* have a dispersal pathway between them. Moreover, there is a critical value 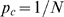, such that for 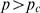 the great majority of the patches in the network become connected to one another by a pathway of edges forming the celebrated “Giant Component” in which there is network-wide connectance [14], [15]. Hence 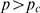 is a necessary condition for a coherent (ER) metapopulation. Conversely for 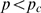, the majority of patches are disconnected. In the latter case, metapopulation persistence is impossible in such poorly connected incoherent network. Although only a simplified system, the ER model makes clear the importance of coherency thresholds and serves to further strengthen the philosophy underlying the EU Habitats Directive for the creation of a coherent Europe-wide network of sites to protect important habitats and species [20].

Taken altogether, the above framework has revealed a number of new insights regarding the determinants of persistence, stability and ecological thresholds in complex metapopulations. We note that a number of results derived here are based on several simplifying assumptions. Thus, at least in the first instance, it was necessary to assume that all patches are identical in terms of their structure and dynamics (a condition that was later relaxed). In addition, it was assumed that the rate of dispersal was the same for all pairs of connected patches. In other applications these model limitations may need to be reckoned with. However, for our purposes, only by fixing patches and metapopulation processes equal, is it possible to isolate exactly how different dispersal networks and their topologies govern persistence. This is in fact a major difference between our work here and that of Armsworth [2]. Although Armsworth also found condition C1 above for a general age-structured metapopulation, his analysis did not result in a quantitative formulation connecting the adjacency matrix 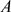, its spectral radius, and its topological features including especially cycles in the network, and their relation to the persistence threshold.

Our analysis was couched both in terms of coarse and fine-scale network features. With regard to the former, at least for random systems, connectivity and heterogeneity in connectivity were found to be two key factors that enhance metapopulation persistence. In terms of more fine-scale features, the existence of critical dispersal routes have been identified, while cycles have been shown to play a prominent functional role, allowing metapopulations to bootstrap themselves into persistence. All of these factors have conservation applications and would translate most readily into principles that aid in the management of marine populations and in the design of networks such as marine protected areas [20]–[22]. In addition these outcomes have obvious applications that cross over into many other scientific contexts including the dynamics of disease epidemics that spread via complex large-scale travel networks, internet traffic and computer virus dynamics or metabolic network analyses [10]–[14].

## Materials and Methods

For a metapopulation of *n* identical patches, the *i*'th patch population may be notated as 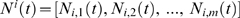 where 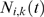 is the number of individuals in the *k*'th age class in year *t*. The model of n-identical age-structured patch populations 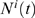 may be written using Kronecker product matrix notation. Let 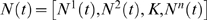, then:
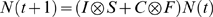
(10)where 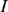 is the 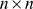 identity matrix , 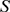 is the lower diagonal matrix with 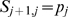 and otherwise zero. 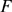 has entries 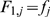 and is otherwise zero. (Note that 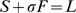.) The above model might also be viewed as a first order approximation to a more general nonlinear metapopulation about the extinction state. After appropriate matrix manipulation, Text S1 shows that the stability of the extinction state depends on the eigenvalues of the stability matrix
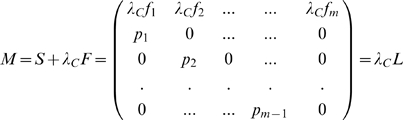
(11)where 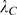 is the spectral radius of the connectivity matrix 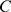. Due to the correspondence between the metapopulation stability matrix 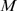 and the Leslie matrix 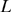 above, the metapopulation (Eqn. 11) is persistent and growing if 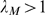, or equivalently 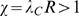 while the extinction state is stable if 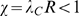. While the threshold is reminiscent of results found for unstructured metapopulations [24], [25] however the inclusion of age-structure and/or network structure makes this is a nontrivial result and a challenge set in Refs. [2], (see Text S1.)

For the particular case 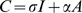, the spectral radius is given by 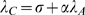 so that 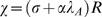.

## Supporting Information

Text S1 The persistence parameter χ. Formulation of age-structured metapopulation model and its mathematical stability criteria.
(0.74 MB PDF)

Text S2 Cycles. Mathematical analysis of the impact of cycles on network stability.
(1.52 MB PDF)

Text S3 Nonidentical patches. Analysis of the metapopulation model for nonidentical patches.
(0.38 MB PDF)
