# Supplementary material for: Connectivity, Cycles, and Persistence Thresholds in Metapopulation Networks
Source: PLoS Comput Biol. 2010 Aug 5;6(8):e1000876. doi: 10.1371/journal.pcbi.1000876 (PMC2916855; doi:10.1371/journal.pcbi.1000876)
Supplement: Text S1 — The persistence parameter χ. Formulation of age-structured metapopulation model and its mathematical stability criteria. (0.74 MB PDF) [file pcbi.1000876.s001.pdf]

Connectivity, Cycles and Persistence Thresholds in Metapopulation Networks

Yael Artzy-Randrup and Lewi Stone

Text S1: The persistence parameter χ

We address the challenge [Ref. 2, 4 and 21 in main text] of developing a framework for analyzing age-structured metapopulations. Begin with a single isolated patch population, and suppose it may be divided into m age-classes as described by the vector $N(t)=\left[N_1(t),N_2(t),...,N_m(t)\right]$ where $N_k(t)$ is the number of individuals in the k'th age class in year t. We suppose the dynamics may be represented by the familiar Leslie matrix equations:

$$N(t+1)=LN(t) \quad \text{where: } L=\begin{pmatrix} \sigma f_1 & \sigma f_2 & \ldots & \ldots & \sigma f_m \\ p_1 & 0 & \ldots & \ldots & 0 \\ 0 & p_2 & 0 & \ldots & 0 \\ . & . & . & . & . \\ 0 & \ldots & \ldots & p_{m-1} & 0 \end{pmatrix} \qquad \text{[S1.1]}$$

and the time-step is one year. Here p_k is the probability that an individual of age (k-1) years survives to age-k. A fraction σ of juveniles from the population that successfully "self-recruit" and return to the population after the dispersal phase, which in marine settings might represent local larvae retention. The parameter f_k represents the fertility of age-class-k individuals, in terms of the average number of juveniles produced in the next generation.

The average number of juveniles produced in the lifetime of a typical individual is given by the reproductive number [Ref. 31 in main text]:

$$R=\sum_{k=1}^{m} f_k \prod_{l=0}^{k-1} p_l \, . \qquad \text{[S1.2]}$$

The condition for population persistence (i.e., a growing population) requires that a typical individual is capable of replacing itself and give rise to at least a single

offspring that successfully recruits back to the population. Thus a growing persisting population requires that the persistence parameter, χ, be larger than 1:

$$\chi = \sigma R > 1 . \qquad \text{[S1.3]}$$

Should $\chi = \sigma R < 1$, all age-classes approach a stable extinction state ($N_i^* = 0$).

Scaling up from the single patch model of eqn. S1.1 consider a network of n age-structured patch-populations, where juveniles disperse between patches. The metapopulation dynamics are given by:

$$N^i(t+1) = S^i N^i(t) + \sum_{j=1}^{n} c_{ij} F^j N^j(t) \qquad \text{[S1.4]}$$

In this notation $N^i(t)$ is the m-dimensional age-class population vector at patch-*i,* and each patch has its own associated survival (S) and fertility (F) matrices specifying the respective p_i and f_i similar to the Leslie formulation in eqn. S1.1.

$$P^i = \begin{pmatrix} 0 & 0 & \dots & \dots & 0 \\ p_1^i & 0 & \dots & \dots & 0 \\ 0 & p_2^i & 0 & \dots & 0 \\ . & . & . & . & . \\ 0 & \dots & \dots & p_{m-1}^i & 0 \end{pmatrix} \text{ and } F^i = \begin{pmatrix} f_1^i & f_2^i & \dots & \dots & f_m^i \\ 0 & 0 & \dots & \dots & 0 \\ 0 & 0 & 0 & \dots & 0 \\ . & . & . & . & . \\ 0 & \dots & \dots & 0 & 0 \end{pmatrix}$$

Dispersal processes between the n-patches are defined in the connectivity matrix $C = (c_{ij})$ whose elements c_{ij} correspond to the proportion of juveniles produced on local population-*j* that are transported and successfully recruit to local population-*i*.

We emphasize that to our knowledge no other study has attempted to solve these $m \times n$ age structured equations. At most approximations may be made that attempt to factor out age-structure, but they are not equivalent systems and their validity will remain questionable [Kaszkurewicz and Bhaya, 2000].

The model of *n*-identical patches [S1.4] may be rewritten in simplified form using Kronecker product matrix notation. Let $N(t)=\left[N^1(t),N^2(t),...,N^n(t)\right]$, then:

$$N(t+1)=(I\otimes S+C\otimes F)N(t) \qquad \text{[S1.5]}$$

where I is the $n\times n$ identity matrix, S is the lower diagonal matrix $S(j+1,j)=p(j)$ that is otherwise zero, and F has entries $F(1,j)=f_j$ but otherwise zeroes. Thus $S+\sigma F=L$ in eqn. [S1.1].

Setting $N(t)=N^*+\delta(t)$ and examining the Jacobian J of eqn. [S1.5] about the extinction equilibrium N^* gives:

$$\delta(T+1)=J\delta(t)=D\left[(I\otimes S+C\otimes F)N(t)\right]\delta(t)=(I\otimes S+C\otimes F)\delta(t)$$

where $D[M]_{ij}=\partial M_{ij}/\partial N_j$.

We use the identity $(C\otimes F)(G\otimes H)=CG\otimes FH$, and assume that C is diagonalizable, i.e., $C=PD_cP^{-1}$, and $D_c=diag(\lambda_i)$ is the diagonal matrix whose elements are the eigenvalues of C. Note that

$$C\otimes F=PD_cP^{-1}\otimes F=PD_cP^{-1}\otimes IFI=(P\otimes I)(D_c\otimes F)(P^{-1}\otimes I).$$

Hence $(P^{-1}\otimes I)(I\otimes S+C\otimes F)(P\otimes I)=I\otimes S+D_c\otimes F$ and the spectral radius of J is the spectral radius of $I\otimes S+D_c\otimes F$, and thus equal to the spectral radius of:

$$M=S+\lambda_C F=\begin{pmatrix} \lambda_C f_1 & \lambda_C f_2 & \ldots & \ldots & \lambda_C f_m \\ p_1 & 0 & \ldots & \ldots & 0 \\ 0 & p_2 & 0 & \ldots & 0 \\ . & . & . & . & . \\ 0 & \ldots & \ldots & p_{m-1} & 0 \end{pmatrix}=\lambda_C L$$

Due to the correspondence between the metapopulation stability matrix $\boldsymbol{M}$ and the Leslie matrix L in [S1.1], namely $M = \lambda_c L$, the metapopulation [S1.5] is persistent and growing if $\lambda_M > 1$ or equivalently $\chi = \lambda_C R > 1$, while the extinction state is stable if

$$\chi = \lambda_C R < 1 \ . \qquad [S1.6]$$

While the criterion is reminiscent [Refs. 24 and 25 in main text] of results found for unstructured metapopulations, however the inclusion of age-structure together with network structure makes this is a nontrivial problem and a challenge set in Ref. 2, 4 and 21 of main text. For the particular case in the text $C = \sigma I + \alpha A$ and the spectral radii satisfy $\lambda_C = \sigma + \alpha \lambda_A$ so that $\chi = (\sigma + \alpha \lambda_A) R$.

<u>**Closed Systems:**</u> It is of interest to consider the special case of "closed" metapopulations in which there are no larvae losses; all larvae are successfully recruited back into the metapopulation. Mathematically this occurs when the connectivity matrix has the property $\sum_{i=1}^{n} c_{ij} = 1$, a property that ensures that the spectral radius of the connectivity matrix C is $\lambda_C = 1$. The criterion for persistence thus becomes: $\chi = R > 1$ and conversely the extinction state is stable if $\chi = R < 1$.

Thus for all closed systems (e.g., the regular networks above), the dispersal structure has no effect whatsoever on the dynamics. That is to say, the stability of the extinction state of the *n*-patch metapopulation is equivalent to the stability of this state in a fully self-recruiting ($\sigma = 1$) single patch, namely $R < 1$.

It is remarkable that for the threshold criteria [S1.6] (or Eqn. 6 in main text), the effects of age structure are ultimately subsumed in the reproductive number R might not appear to be otherwise influential. However, the effects of age-structure become more prominent under more complex migration schemes, for example, between different age-classes from different patches, and ontogenetic shifts in habitat use that are life history dependent [Ref. 21 in main text]. The model can be extended for

these situations by using the full equations [S1.4] above and, for example, adding elements to the matrix F. Text S3 deals with other techniques for exploring this possibility further.

Asymmetric Networks: Metapopulation models usually assume that dispersal is a function of distance and not directional, making the connectivity matrix symmetric. Yet many real ecological networks are asymmetric, which according to recent simulation studies [Ref. 36 in main text], has a negative effect on metapopulation persistence [Ref. 37 in main text]. Compare a random ER model to one modified to have asymmetric directed connections. The latter has the same number of patches and randomly distributed but now directed edges. The average number of connections per patch is $\bar{r}$, but only $\bar{r}/2$ disperse out of each patch. The spectral radius of the directed network is half [Ref. 34 in main text] that of its undirected counterpart, and thus $\chi = \left(\sigma + \bar{r}\,\alpha/2\right)R$, which can be far smaller than (Eqn. 8 in main text) $\chi = \left(\sigma + \bar{r}\alpha\right)R$ in the text. Therefore asymmetry should be expected to have a negative effect on persistence. In the text we provide a complementary explanation as to why symmetry promotes persistence due to the presence of "cycles."

REFERENCE:

Kaszkurewicz E and Bhaya A (Eds.) Matrix diagonal stability in systems and computation. *pp*. 267. Birkhäuser, Boston (2000)
