# Supplementary material for: Connectivity, Cycles, and Persistence Thresholds in Metapopulation Networks
Source: PLoS Comput Biol. 2010 Aug 5;6(8):e1000876. doi: 10.1371/journal.pcbi.1000876 (PMC2916855; doi:10.1371/journal.pcbi.1000876)
Supplement: Text S2 — Cycles. Mathematical analysis of the impact of cycles on network stability. (1.52 MB PDF) [file pcbi.1000876.s002.pdf]

Connectivity, Cycles and Persistence Thresholds in Metapopulation Networks

Yael Artzy-Randrup and Lewi Stone

Text S2: Cycles

Basic definitions

Consider a metapopulation network on n patches and denote $A = \{a_{ij}\}$ as its adjacency matrix such that for a directed dispersal route from patch-*j* to patch-*i*, $a_{ij} = 1$, and otherwise, $a_{ij} = 0$. We assume that there are no self-loops and thus for all *i*, $a_{ii} = 0$.

We define a **cycle** as a closed directed path on a network, where the nodes and links may be repeated along the path, and a **simple cycle** of length m as a cycle that has exactly m nodes and m links. Following these definitions we divide a network into **cyclic components**, such that each component is characterized as being one of the following:

a) A single node component: a component containing exactly one node and zero links, where the node is not a member of any cycle in the network.

b) A simple cycle component: a component containing a group of links and nodes which are members of a simple cycle, as defined above, with an additional assumption that all the nodes in this component are members only of this cycle and of no other in the network.

c) A complex cycle component: a component containing a group of links and nodes members of a cycle that is not simple, under the assumption that all nodes in this component are members only of this cycle and of no other in the network.

Note that this division may be seen as a partition of the nodes into groups, such that each node is a member of exactly one of the three above types of cyclic components. However, this is not a partition of the links, since each link belongs to at most one component, and it is possible that there are links that are not members of any component. These links are defined as **lonely links**.

Some properties of cyclic components

1) A simple cycle component of size k has exactly k nodes and k links and contains exactly one closed directed path of length k. A complex cycle component of size k has k nodes and at least k+1 links and contains at least two closed directed paths, all of which may be shorter than k.

2) From the definitions above, a directed path exists between each pair of nodes in simple and complex cycle components. This includes a non-empty directed path going from each node to itself.

3) The addition of a link to a simple cycle component of size k turns the component in to a complex cycle component of size k that has k nodes and k+1 links. This new component contains exactly two closed directed paths, one of length k and the other of length $m<k$.

Finding the eigenvalues

To calculate the characteristic polynomial of matrix A, we define matrix B as: $\boldsymbol{B} = \boldsymbol{A} - \lambda \boldsymbol{I}$, where I is the nxn identity matrix. Using the Leibniz formula, the characteristic polynomial of A, p_A, can be written as:

$$p_A(\lambda) = |\boldsymbol{B}| = \left| \begin{pmatrix} b_{1,1} & \cdots & b_{1,n} \\ \vdots & \ddots & \vdots \\ b_{m,1} & \cdots & b_{m,n} \end{pmatrix} \right| = \sum_{\sigma \in S_n} (\operatorname{sgn} \sigma) \prod_{i=1}^{n} b_{i,\sigma(i)} \qquad [S2.1]$$

where σ is a permutation on $\{1,...,n\}$, and S_n is the group of such permutations (in total $|S_n| = n!$). $\operatorname{sgn}\sigma$ equals either 1 or -1, depending on whether the permutation is even or odd, respectively. Also note that for $i \neq j$, $b_{i,j}$ equals either 0 or -1, and on the diagonal $b_{i,i} = -\lambda$.

For a typical permutation, $\sigma \in S_n$, it is straightforward to show that the product $\prod_{i=1}^{n} b_{i,\sigma(i)}$ must equal either 0 or $(-\lambda)^{n-m}$, where m is the number of indices that fulfill $i \neq \sigma(i)$.

Clearly, for the *m-n* cases when $i = \sigma(i)$, $b_{i,\sigma(i)} = -\lambda$. However, for the remaining *m* cases, when $i \neq \sigma(i)$, we find that the product $\prod_{k=1}^{m} b_{j_k,\sigma(j_k)}$ equals 1^m if and only if for all j_k's in this product there exists a link going from $\sigma(j_k)$ to j_k (that is, $a_{j_k,\sigma(j_k)} = 1$), and otherwise this product equals 0.

Because a permutation can be decomposed into a product of disjoint permutation cycles (PC), such that PCs of length 1 are associated with the cases where $i = \sigma(i)$, and PCs longer than 1, with the cases where $i \neq \sigma(i)$, we find that $\prod_{i=1}^{n} b_{i,\sigma(i)} = (-\lambda)^{n-m}$ if and only if for all $i \neq \sigma(i)$ the link going from $\sigma(j_k)$ to j_k in the network is on a cycle, and otherwise, $\prod_{i=1}^{n} b_{i,\sigma(i)} = 0$.

Implications

1) **The eigenvalues λ_i of an adjacency matrix A of a network, which has no cycles (i.e., all of the cyclic components are *single node components*), are all zero i.e., $\lambda_i = 0$. Thus, a network with no cycles cannot persist.**

This is because when there are no cycles, from the definitions above, the characteristic polynomial of A is equal to $p_A(\lambda) = \prod_{i=1}^{n} b_{i,i} = (-\lambda)^n$, and the eigenvalues all equal 0.

In a metapopulation network that is completely without cycles, all juveniles and any of their eventual descendants fail to recruit back to their patch of origin – they never "return home" [Ref. 3 in main text]. When there are no cycles, the adjacency matrix $\boldsymbol{A}$ must have spectral radius $\lambda_{\boldsymbol{A}} = \mathbf{0}$. Hence in the absence of self-recruitment $(\sigma = 0)$, the persistence parameter $\chi = (\sigma + \lambda_{\boldsymbol{A}}\varepsilon)\boldsymbol{R} = \mathbf{0}$ is less than unity and so the metapopulation is unable to persist. A stable extinction state is expected.

For metapopulations with self recruitment but without any other cycles, there is no advantage to dispersal. Self-recruitment implies that a proportion σ of juveniles are retained in the patch. As there are no other cycles, all other juveniles (and/or their descendants) fail to return home and the spectral radius remains $\lambda_{\boldsymbol{A}} = \mathbf{0}$ and $\chi = \sigma\boldsymbol{R}$. Thus the criterion for the entire metapopulation to persist, $\chi = \sigma\boldsymbol{R} > \mathbf{1}$, is precisely the same as the criterion for a single self-recruiting patch to persist (see eqn. 3 in main text). Consequently there is no advantage for dispersal if there are no cycles and larvae fail to "return home" [Ref. 3 in main text].

2) **Noting C as the adjacency matrix for the network after removing all of the lonely links, the eigenvalues of adjacency matrices A and C are equal. Thus, links which are not part of a cycle do not have any role in determining the persistence of a network.**

To see this, take a link in the network going from j to i ($j \neq i$), such that $a_{ij} = 1$ and following from that, $b_{ij} = -1$. Assume this link is not a member of any cycle. Without loss of generality, take a permutation $\sigma' \in S_n$, where $\sigma'(j) = i$. When decomposing σ' into a product of disjoint PCs, $\sigma'(j) = i$ is a member of a PC which must be

longer than one because $j \neq i$. However, because we assumed that a_{ij} is not a member of any cycle, there must be another permutation $\sigma'(j^*) = i^*$ in this PC such that $a_{i^*,j^*} = 0$. Thus $\left(\operatorname{sgn}\sigma'\right)\prod_{i=1}^{n} b_{i,\sigma'(i)} = 0$, and so there is no contribution of this term to the RHS sum in equation S2.1, making $p_{A_M}(\lambda) = p_C(\lambda)$.

Example 1: The characteristic polynomial of the adjacency matrix of both of the networks is equal: $p_A(\lambda) = \lambda^8(\lambda^3 - 1)$, and the maximum eigenvalue is 1.

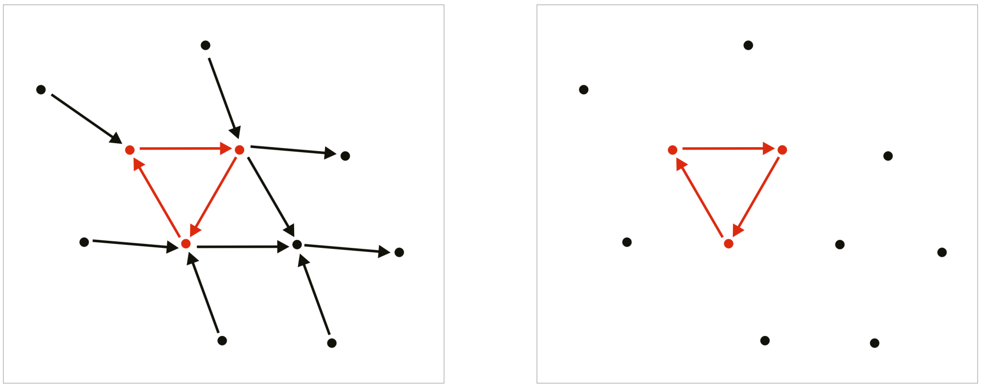

3) The maximum eigenvalue of an adjacency matrix *A*, which has exactly one simple cycle component and no complex cycle components, is real and equal to 1.

From the definitions above [S2.1] the characteristic polynomial is $p_A(\lambda) = (-\lambda)^n + (-\lambda)^{n-m}(-1)^{m+1} = (-1)^n \lambda^{n-m}(\lambda^m - 1)$, where m is the length of the simple cycle. Thus, there are *n-m* eigenvalues that equal zero, and the rest are the m roots of unity ($l = 1 \ldots m$: $\lambda_l = e^{i\frac{2\pi l}{m}}$). Example 1 in the previous section demonstrates this.

4) **Taking each cyclic component as a network, the characteristic polynomial of the adjacency matrix of:**

a) A single node component is of the form:

$$p_I(\lambda) = -\lambda \qquad [S2.2]$$

b) A simple cycle component of size *n*:

$$p_{II}(\lambda) = (-1)^n (\lambda^n - 1) \qquad [S2.3]$$

c) A complex cycle component of size *n*:

$$p_{III}(\lambda) = (-1)^n \begin{cases} \lambda^n + \\ \lambda^{n-2}(-D_2) + \\ \lambda^{n-3}(-D_3) + \\ \lambda^{n-4}(-D_4 + D_{2,2}) + \\ \lambda^{n-5}(-D_5 + D_{3,2}) + \\ \lambda^{n-6}(-D_6 + D_{4,2} + D_{3,3} - D_{2,2,2}) + \\ \lambda^{n-7}(-D_7 + D_{5,2} + D_{4,3} - D_{3,2,2}) + \\ \lambda^{n-8}(-D_8 + D_{6,2} + D_{5,3} + D_{4,4} - D_{4,2,2} - D_{3,3,2} + D_{2,2,2,2}) + \\ \lambda^{n-9}(-D_9 + D_{7,2} + D_{6,3} + D_{5,4} - D_{5,2,2} - D_{4,3,2} - D_{3,3,3} + D_{3,2,2,2}) + \\ \vdots \end{cases}$$

[S2.4]

where D_i is the number of simple cycles of length *i*. $D_{i,j}$ is the number of couples of simple cycles, where one is of length *i* and the other of length *j* which don't share any links or nodes. $D_{i,j,k}$ is the number of triplets of simple cycles, where one is of length *i*, the second of length *j* and the third of length *k*, which don't share any links or nodes, and so on. Note that the sum of the indexes in *D* for the coefficients of λ^{n-i} equal *i*, and that the sign of *D* is determined by the number of indexes (e.g., $D_{i,j}$ has an even

number of indexes and will always have a positive sign, while $D_{i,j,k}$ has an odd number of indexes and will always have a negative sign).

[S2.2] and [S2.3] were derived as explained in sections 1 and 3, and [S2.4] is directly obtained by the Leibniz formula in [S2.1].

Example 3 in section 6 demonstrates these formulas.

5) The maximum eigenvalue of an adjacency matrix for a complex cycle component of k nodes is larger than 1, and reaches its maximum at $(k-1)$ when the component is fully connected (i.e., when the component has $k(k-1)$ links).

Take G to be the group of nodes and links of a complex cycle component of size k. Because this component has at least k+1 links, there exists at least one node which has at least two links pointing out from it. Take one of these nodes, mark it as g_1, and choose a non-empty subgroup of nodes and links which define a closed directed path going from this node to itself. We name this subgroup G_1 with size k_1 ($k_1 \le k$), and note that G_1 is a simple cycle (G_1 contains k_1 nodes and k_1 links). Taking a second link pointing out of g_1, define a new group G_2 that consists of the nodes and links in G_1 and this new link. If this link does not point to any of the nodes in G_2, add the node it is pointing to, to G_2. It is possible to continue adding nodes and links of a directed path (without repeats) until a link is found which points to one of the original nodes taken from G_1. Mark the size of G_2 as k_2 such that the number of nodes is k_2 ($k_1 < k_2 \le k$), and the number of links is k_2+1. Note that this is a complex cycle component that consists of exactly two simple cycles that are of length k_3 and k_4 ($k_4 \le k_3 \le k_2$).

From section 4, the characteristic polynomial of the adjacency matrix of G_1 is of the form: $p_{II}(\lambda) = (-1)^{k_1}(\lambda^{k_1}-1)$, and its maximum eigenvalue is 1. Because G_2 consists of exactly two simple cycles, the characteristic polynomial of its adjacency matrix is

one of three forms: *i*) if $k_3 = k_4$: $p_{III}(\lambda) = (-1)^{k_2} \lambda^{k_2 - k_3} (\lambda^{k_3} - 2)$, *ii*) if $k_3 = k_2$: $p_{III}(\lambda) = (-1)^{k_2} (\lambda^{k_2} - \lambda^{k_2 - k_4} - 1)$, or else *iii*) $p_{III}(\lambda) = (-1)^{k_2} \lambda^{k_2 - k_3} (\lambda^{k_3} - \lambda^{k_3 - k_4} - 1)$, non of which have a root = 1.

Because $G_1 \subset G_2 \subseteq G$ their adjacency matrices $A_1 < A_2 \leq A$ (respectively), and as such the spectral radii (i.e., the maximal eigenvalues) fulfill $\sigma(A_1) \leq \sigma(A_2) \leq \sigma(A)$. However, because $\sigma(A_1) = 1$, and we have shown 1 is not a root of the characteristic polynomial of A_2, we find that $1 < \sigma(A)$, such that the maximal eigenvalues of complex cycle components must be larger than 1.

Along these lines it is clear that with the addition of links to a complex cycle component, the maximal eigenvalue increases. When the component is fully connected (i.e., is a clique), the adjacency matrix has values of 1's in all cells of the adjacency matrix except for the diagonal. By induction on *k* it is possible to show that the characteristic polynomial is $p_{III}(\lambda) = (-1)^k (\lambda - k + 1)(\lambda + 1)^{k-1}$, and the maximum eigenvalue equals *k*-1.

Example 2: Sampling cyclic components ranging in size from 1 node to 30, we plotted the maximum eigenvalue of each component as a function of the link density (i.e., the number of links divided by the number of nodes). The x-axis is the ratio of links to nodes, and the y-axis is the maximum eigenvalue. We see that as the density of links in a cyclic component grows, so does the maximum eigenvalue. It is clear to see that for single node components (where the number of links is 0, in green), the maximum eigenvalue is always 0, and that for simple cycle components (where the number of links equals the number of nodes, in red), the maximum eigenvalue is always 1. For complex cycle components (where the number of links is larger than the number of nodes, in blue) we see that the maximum eigenvalues are always larger than 1, up to the maximum point where the number links is maximal.

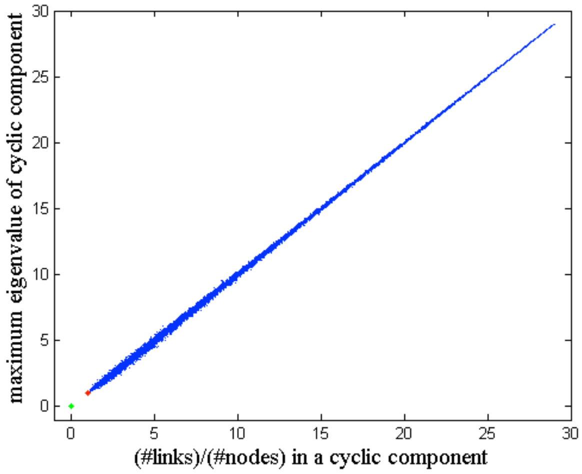

6) The characteristic polynomial of adjacency matrix A can be written as a product of the characteristic polynomials of each of the cyclic components in the network:

$$p_A(\lambda)=\left(\prod_{i=1}^{k_I} p_{I,i}(\lambda)\right)\left(\prod_{i=1}^{k_{II}} p_{II,i}(\lambda)\right)\left(\prod_{i=1}^{k_{III}} p_{III,i}(\lambda)\right) \qquad \text{[S2.5]}$$

where k_I is the number of single node components, k_{II} is the number of simple cycle components and k_{III} is the number of complex cycle components. p_I , p_{II} and p_{III} are the polynomials as defined in section 4.

Thus, the maximum eigenvalue of A is determined by the cyclic component with the maximum eigenvalue.

To see this, note that from the properties of isomorphism of networks, we may assume that the nodes of the network are ordered in a way that indices of the nodes within each cyclic component are adjacent. As such, matrix $\boldsymbol{C}$ (as defined in section 2) is a block diagonal matrix of the form:

$$\boldsymbol{C}=\begin{pmatrix} \boldsymbol{C_1} & & 0 \\ & \ddots & \\ 0 & & \boldsymbol{C_k} \end{pmatrix}$$

where $\boldsymbol{C}_i$ are the adjacency matrices of each cyclic component in the network. From the properties of block diagonal matrices we know that: $|\boldsymbol{C}| = \prod_{i=1}^{k} |\boldsymbol{C}_i|$. Thus,

$$p_A(\lambda) = p_C(\lambda) = |\lambda \boldsymbol{I} - \boldsymbol{C}| = \prod_{i=1}^{k} |\lambda \boldsymbol{I} - \boldsymbol{C}_i| = \prod_{i=1}^{k} p_{C_i}(\lambda).$$

Example 3:

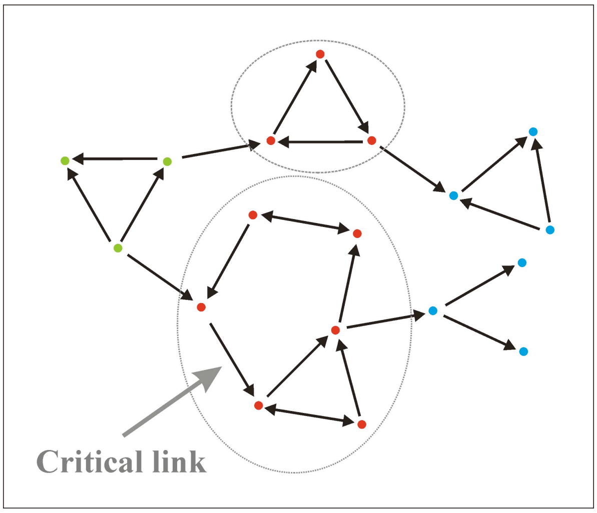

This metapopulation network has 18 nodes (patches) and 24 links: 9 single node components, 1 simple cycle component consisting of 3 nodes and 3 links, 1 complex cycle component consisting of 6 nodes and 9 links, and 12 lonely links. The characteristic polynomial is:

$$p_{A_M}(\lambda) = \underbrace{\left((-\lambda)^9\right)}_{9 \text{ single node comp}} \cdot \underbrace{\left((-1)^3\left(\lambda^3 - 1\right)\right)}_{\substack{1 \text{ simple cycle comp} \\ \text{of size } 3}} \cdot \underbrace{\left((-1)^6\left(\lambda^6 - 2\lambda^4 + \lambda^2 - \lambda - 1\right)\right)}_{\substack{1 \text{ complex cycle comp} \\ \text{of size } 6}}$$

The characteristic polynomial of the complex component is derived as follows:
The size of this component is 6. This component contains 2 simple cycles of length 2, so $D_2 = 2$. These two simple cycles don't share any links or nodes, so $D_{2,2} = 1$. There is one simple cycle of size 5 ($D_5 = 1$), and one simple cycle of size 6 ($D_6 = 1$).

From [S2.4] we find: $p_{III}(\lambda)=(-1)^6(\lambda^6-2\lambda^4+\lambda^2-\lambda-1)$.

The maximum eigenvalue of the single node components is 0, the maximum eigenvalue of the simple cycle component is 1 and the maximum eigenvalue of the complex cycle component is 1.4433. Indeed this is the maximum eigenvalue of the entire network, and we see that it is determined by the cyclic component with the maximal ratio between links to nodes. Note that the maximum eigenvalue (1.4433) is approximately equal to the ratio, 9:6=1.5, as expected from section 5.

If the link marked "critical link" were to be remover we would be left only with simple cyclic components and the new characteristic polynomial would be:

$$p_{A_M}(\lambda) = \underbrace{\left((-\lambda)^{11}\right)}_{11\ \textit{single node comp}} \cdot \underbrace{\left((-1)^3(\lambda^3-1)\right)}_{\substack{1\ \textit{simple cycle comp} \\ \textit{of length}\ 3}} \cdot \underbrace{\left((-1)^2(\lambda^2-1)\right)^2}_{\substack{2\ \textit{simple cycle comp} \\ \textit{of length}\ 2}} = 0$$
