# Supplementary material for: Connectivity, Cycles, and Persistence Thresholds in Metapopulation Networks
Source: PLoS Comput Biol. 2010 Aug 5;6(8):e1000876. doi: 10.1371/journal.pcbi.1000876 (PMC2916855; doi:10.1371/journal.pcbi.1000876)
Supplement: Text S3 — Nonidentical patches. Analysis of the metapopulation model for nonidentical patches. (0.38 MB PDF) [file pcbi.1000876.s003.pdf]

Connectivity, Cycles and Persistence Thresholds in Metapopulation Networks

Yael Artzy-Randrup and Lewi Stone

Text S3: Nonidentical Patches

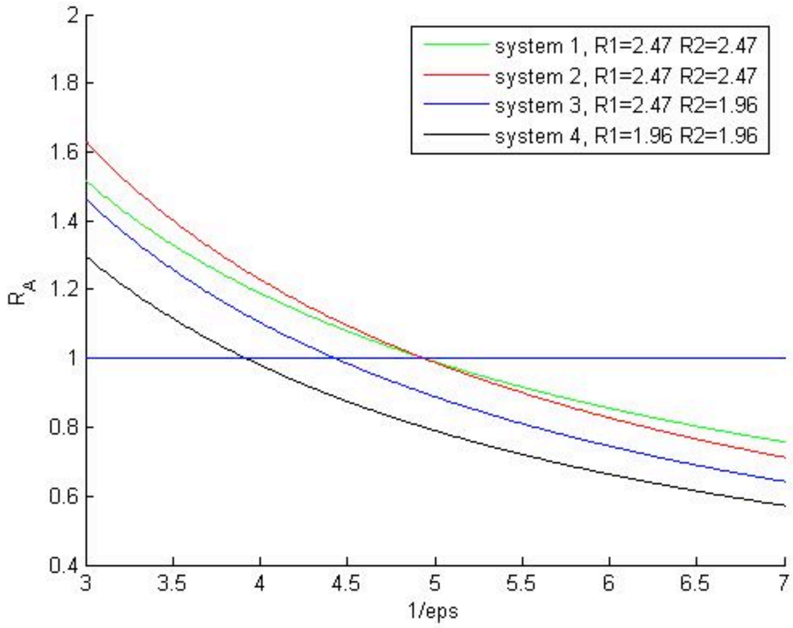

Consider the extinction criteria for a metapopulation consisting of two connected and nonidentical patches in which patch-1 dominates (R_1=2.47 >R_2=1.96). The blue line plots the eigenvalue of the Jacobian matrix as a function of ($1/\alpha$) where α is the dispersal.

As described in the text construct a new metapopulation consisting of 2-copies of the dominating patch-1 retaining the original network structure. Thus both patches have R_1=R_2=2.47.

The persistence parameter is $\chi = 2\alpha R_1 = 4.94\alpha$. The red line plots the plots the largest eigenvalue of the Jacobian matrix, obtained numerically, as a function of ($1/\alpha$) where e is the dispersal. Stable extinction occurs when this eigenvalue is less than one in magnitude, corresponding exactly to the prediction $\chi < 1$ or $1/\alpha < 4.97$.

We see that if the red line (identical patches) has values smaller than 1 the blue line (nonidentical patches) will also and stable extinction will occur. This is the basis for understandingextinction from a study of the metapopulation's dominating patch alone.

Now consider persistence conditions for a metapopulation consisting of two connected and nonidentical patches in which patch-2 is subordinate (R_1=2.47 >R_2=1.96). Again, the blue line plots the eigenvalue of the Jacobian matrix as a function of ($1/\alpha$) where e is the dispersal. As described in the text construct a new metapopulation consisting of 2-copies of the subordinate patch-2 retaining the original network structure. Thus both patches have R_1=R_2=1.96.

The persistence parameter is $\chi = 2\alpha R_1 = 3.92\alpha$. The black line plots the plots the largest eigenvalue of the Jacobian matrix, obtained numerically, as a function of ($1/\alpha$) where e is the dispersal

. Persistence occurs when this eigenvalue is greater than one in magnitude, corresponding exactly to the prediction $\chi > 1$ or $1/\alpha > 3.92$.

We see that if the black line (identical patches) has values greater than unity the blue line (nonidentical patches) will also and persistence will occur. This is the basis for understanding persistence from a study of the metapopulation's subordinate patch alone.

Mathematical analysis for nonidentical patches: Consider a set of n-nonidentical patches in which patch-1 dominates (see text). As described in the text construct a new metapopulation consisting of *n*-copies of the dominating patch-1 having the original network structure. Associated to this metapopulation is the nonnegative matrix $J' = \left(I \otimes S + C \otimes F\right)$ as in Supplementary Information 1. Assume that the spectral radius $\rho\left(J'\right) < 1$ so that the extinction state is stable. As the matrix J' is positive, it is Hadamard stable (Kaszkurewicz & Bhaya 1999, p.70) which implies that for any matrix $T = \left(t_{ij}\right)$and $0 \le t_{ij} \le 1$, the entry wise (Hadamard)

product $J = T \circ J'$ also has spectral radius $\rho(J) < 1$ It follows that the metapopulation dynamics of the *n*-nonidentical patches must satisfy the modified equations:

$$N(t+1) = T(I \otimes S + C \otimes F)N(t) = J\,N(t)$$

and have a stable extinction state when $\rho(J) < 1$ A sufficient condition for stable extinction state of the nonidentical patches is thus $\rho(J') < 1$ or equivalently $\chi \le 1$. (Note again that here J' is to be associated with the n-copies of dominating patch-1.) Moreover the system stability is robust to time-varying delays (Theorem 3.6.2(i), Kaszkurewicz and Bhaya, 1999).

A similar argument shows the converse result for persistence of *n*-nonidentical patches based on the subordinate patch.

1) **Extinction:** Without loss of generality suppose patch-1 of the metapopulation "dominates" in the sense that it has the highest fertility parameters and highest survival rates, and thus the highest net reproductive effort (R_1). Now construct a metapopulation comprised of *n*-identical copies of dominating patch-1, retaining the original directed or undirected network structure. Test whether the extinction state of these *n*-identical patches is stable. If so, we show that the original heterogeneous metapopulation of *n*-nonidentical patches is also unable to persist. That is, the dominant patch population, may be used as a guide for determining the fate of the larger heterogenous metapopulation (see figure 5 in text).

2) **Persistence:** Along similar lines, suppose patch-1 is the weakest in the sense that it has the lowest fertility parameters and lowest survival rates. If the network of *n*-identical copies of patch-1 is able to persist, then the original heterogeneous metapopulation must also persist (see figure 5 in text).

REFERENCE:

Kaszkurewicz E and Bhaya A (Eds.) Matrix diagonal stability in systems and computation. *pp*. 267. Birkhäuser, Boston (2000)
